# Human leukocyte antigen pharmacogenetics in infectious diseases: anti-infective drug toxicity and host immune response

**DOI:** 10.3389/fphar.2026.1846785

**Published:** 2026-06-04

**Authors:** Simone Negrini, Stefania Nicola, Iuliana Badiu, Anna Quinternetto, Ilaria Vitali, Luca Lo Sardo, Luisa Brussino

**Affiliations:** 1 Allergy and Immunology Unit, Mauriziano Hospital, Turin, Italy; 2 Department of Medical Sciences, University of Turin, Turin, Italy

**Keywords:** adverse drug reactions, anti-infectives, drug hypersensitivity, human leukocyte antigen, infection outcomes, pharmacogenetics, precision medicine, vaccine response

## Abstract

Human leukocyte antigen (HLA) polymorphisms are central to anti-infective pharmacogenetics and to inter-individual variability in infection outcomes and vaccine responses, but clinical relevance depends on whether an association changes treatment or prevention decisions. This narrative review is organized around two complementary pillars: first, severe, typically T cell–mediated adverse drug reactions to anti-infective agents, where HLA can support prevention when genetic effect, phenotype precision, and therapeutic alternatives converge; and second, selected, replicated HLA-region associations with infection outcomes or vaccine immunogenicity, where biological effects may be robust yet individual-level clinical translation is often limited. Within the adverse drug reaction pillar, the clearest preventive paradigm remains HLA-B*57:01-guided abacavir prescribing, with additional high-signal examples including dapsone hypersensitivity and flucloxacillin-induced liver injury that illustrate how large effect sizes do not always justify routine screening. Within the infection and vaccine pillar, HIV, HCV, and hepatitis B vaccine response provide the strongest evidence that HLA shapes clinically relevant host-response heterogeneity. Across both domains, the key pharmacological distinction is between mechanistically persuasive associations and those that are sufficiently robust, transportable, and decision-relevant to change practice.

## 1 Introduction

Human leukocyte antigen (HLA) molecules are cell-surface glycoproteins encoded within the major histocompatibility complex (MHC) on chromosome 6p21.3. Their primary immunological function is to present peptide antigens to T lymphocytes, thereby shaping adaptive immune recognition in health and disease (Neefjes et al., 2011; Rock et al., 2016).

**FIGURE 1:**
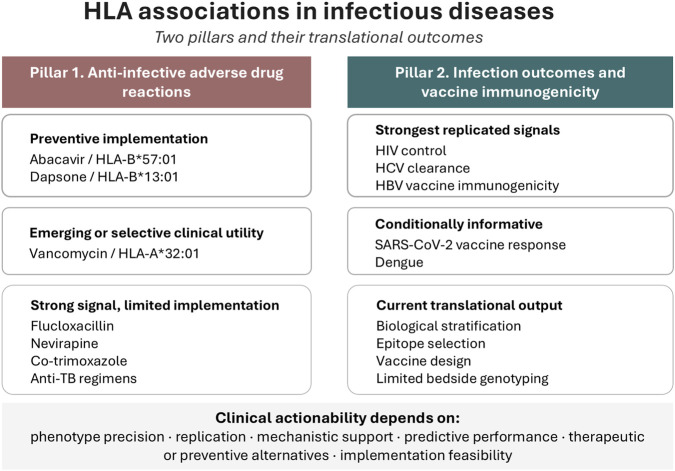
Conceptual overview of the two-pillar structure of the review and the translational gradient of HLA associations in infectious-disease pharmacogenetics. Legend. The figure summarizes the two-pillar architecture of the review and their different translational trajectories. In Pillar 1, selected HLA associations affecting anti-infective adverse drug reactions have sufficient predictive and practical value to support prevention or selective clinical implementation. In Pillar 2, even strong and replicated HLA associations are more often informative for biological stratification, epitope selection, or vaccine design than for routine individual-level bedside genotyping. Examples are illustrative rather than exhaustive and are positioned according to their current translational status as discussed in the main text.

The same HLA-dependent antigen-presentation machinery can support either protective anti-pathogen immunity or maladaptive drug-specific immunopathology, depending on the antigenic context (Neefjes et al., 2011; Rock et al., 2016). In this narrative review, we focus on HLA-mediated immune variability that is most relevant to infectious-disease pharmacology: (i) severe, typically T cell–mediated adverse drug reactions to anti-infective agents (Pillar 1) and (ii) selected, replicated HLA-region associations with infection outcomes or vaccine immunogenicity (Pillar 2). Throughout, we prioritize phenotype rigor, replication, and clinical actionability in pharmacological terms (absolute risk, test performance, and availability of prescribing alternatives). Host genetic variants that primarily influence pharmacokinetics (drug metabolism or transport) are outside the scope of this HLA-centric review. The overall structure of the review and the translational gradient across its two pillars are summarized in Figure 1.

A defining feature of HLA biology—and the reason HLA is repeatedly implicated in infectious diseases, vaccination, and drug safety—is extreme genetic diversity concentrated in peptide-binding regions (Penn et al., 2002; Wegner et al., 2004). Polymorphic residues within peptide-binding grooves yield allele-specific differences in binding pockets and therefore in the repertoire of peptides presented to T cells (Abualrous et al., 2021). This diversity is extensive: the WHO-recognized IPD-IMGT/HLA Database contains tens of thousands of alleles across multiple genes recognized by the WHO Nomenclature Committee for Factors of the HLA System (Barker et al., 2026; Robinson et al., 2020). HLA nomenclature encodes progressively finer allele resolution using up to four colon-separated fields (e.g., HLA-B*57:01:01:01), and two-field resolution (e.g., HLA-B*57:01) is typically the minimum practical level for clinically interpretable pharmacogenomic associations and routine testing (Marsh et al., 2010; Hurley, 2021; Profaizer and Kumanovics, 2018). Imputation approaches can also infer HLA alleles and amino-acid residues from SNP data (Jia et al., 2013). However, imputation performance is reference-panel dependent and may be less reliable in ancestrally diverse or admixed populations, which can affect the transferability of HLA-region associations across settings.

HLA diversity is also strongly structured by ancestry and geography, with substantial between-population variation in allele and haplotype frequencies and extensive linkage disequilibrium across the extended MHC, generating conserved, population-specific haploblocks (de Bakker et al., 2006; Solberg et al., 2008; Traherne, 2008). Resources such as the Allele Frequency Net Database (AFND) compile frequency data at scale; in its 2020 update, AFND reported >1600 populations from >10 million healthy individuals, supporting analyses of highly polymorphic immune loci and their global distributions (González-Galarza et al., 2020). These population features have direct translational implications: the same HLA “risk allele” can differ in frequency, linkage disequilibrium context, and apparent penetrance across ancestries, which can shift observed effect estimates and predictive values and complicate implementation across diverse settings (de Bakker et al., 2006; González-Galarza et al., 2020; Acosta-Monterrosa et al., 2026; Quiñones et al., 2026).

HLA-restricted ADRs represent the most mature and actionable application. The paradigmatic example is abacavir hypersensitivity, where prospective HLA-B*57:01 screening in a randomized trial eliminated immunologically confirmed reactions and enabled routine pre-prescription testing (Mallal et al., 2008; Martin et al., 2014). Beyond abacavir, robust associations exist for other agents, including flucloxacillin drug-induced liver injury (DILI), where HLA-B*57:01 confers a very large genetic risk (Daly et al., 2009), and dapsone-induced cutaneous adverse drug reactions and hypersensitivity syndrome, where HLA-B*13:01 shows a strong association supported by meta-analysis (pooled OR 43.0, 95% CI 24.0–77.2) (Zhang et al., 2013; Tangamornsuksan and Lohitnavy, 2018). However, even large genetic effects do not automatically justify population screening because actionability also depends on phenotype incidence, severity, diagnostic clarity, and availability of therapeutic alternatives (Pirmohamed et al., 2015; Kloypan et al., 2021). In many HLA-associated phenotypes, carriage of the risk allele is necessary but not sufficient, implying that immune context, co-factors, and non-HLA host variation modulate clinical expression (Pirmohamed et al., 2015; Kloypan et al., 2021).

Several translational concepts recur throughout this review and warrant explicit clarification at the outset. Absolute risk refers to the observed probability of an event in exposed individuals or carriers, whereas relative effect measures (such as odds ratios or hazard ratios) describe the strength of association but do not by themselves indicate clinical usefulness. Positive predictive value (PPV) reflects the probability that a test-positive individual will develop the phenotype of interest, whereas negative predictive value (NPV) reflects the probability that a test-negative individual will remain free of that phenotype; both depend not only on test performance but also on phenotype incidence. Phenotype precision is equally critical, because broad or inconsistently defined syndromes can dilute true associations. In HLA studies, ancestry effects and linkage disequilibrium within the MHC region further influence transportability and causal interpretation, since allele frequencies, haplotypic structure, and correlated variants may differ substantially across populations.

Literature search and scope: We performed a targeted narrative literature search in PubMed and Embase through March 2026. The search was structured around two predefined domains: (i) HLA-associated adverse drug reactions to anti-infective agents, including severe cutaneous adverse reactions, DRESS/drug-induced hypersensitivity syndrome, and drug-induced liver injury; and (ii) HLA-region associations with infection outcomes or vaccine immunogenicity. Search terms were combined iteratively around HLA, anti-infective drugs, hypersensitivity, severe cutaneous adverse reactions, drug-induced liver injury, infection susceptibility or control, and vaccine response. Because the aim of the review was interpretive synthesis rather than exhaustive systematic coverage, no formal PRISMA workflow or systematic-review style inclusion/exclusion pipeline was applied. Instead, studies were prioritized according to phenotype rigor, independent replication, mechanistic relevance, and clinical decision relevance. For clinically mature associations, we preferentially considered primary studies with the greatest translational value, including prospective or randomized studies, implementation studies, high-quality meta-analyses, and relevant guidelines or regulatory documents. For emerging or non-actionable associations, we prioritized replicated association studies and mechanistic primary literature over isolated signals without replication. Evidence was synthesized narratively in order to compare biological plausibility with current clinical actionability across heterogeneous phenotypes and study designs. When findings were conflicting, inconsistent across ancestries, or based on heterogeneous endpoints, interpretive emphasis was placed on studies with stronger phenotype adjudication, independent replication, and clearer translational relevance.

## 2 HLA-associated adverse drug reactions to anti-infective agents

### 2.1 Conceptual framework for HLA-mediated adverse drug reactions to anti-infectives

Severe anti-infective adverse drug reactions (ADRs) are often HLA-restricted, yet the initiating molecular event is not uniform across drugs or phenotypes. A central mechanistic question is how small-molecule drugs, far smaller than canonical peptide ligands, can nevertheless engage the HLA–peptide–TCR axis and trigger antigen-specific T-cell activation (Pirmohamed et al., 2015). The current evidence supports a small set of complementary models that converge on the same endpoint: drug-dependent presentation of antigenic determinants capable of triggering T-cell activation (Pirmohamed et al., 2015). This “many roads to the same phenotype” framework is clinically useful because it explains why the same clinical syndrome can arise from different drug classes, why a single drug may engage more than one pathway, and why even strong HLA risk alleles usually show incomplete penetrance. Recent frameworks increasingly favor endotypes—mechanistically defined categories anchored in HLA-restricted antigen presentation and downstream effector programs—over purely phenotype-based labels, especially for heterogeneous entities such as DRESS (Drug Reaction with Eosinophilia and Systemic Symptoms) and SCAR (Severe Cutaneous Adverse Reactions) (Mayorga et al., 2025; Kardaun et al., 2013; Bastuji-Garin et al., 1993; Sidoroff et al., 2007; Shiohara and Mizukawa, 2019). The main dimensions of clinical actionability are summarized in Table 1, and the major mechanistic models and their typical experimental readouts are summarized in Table 2.

**TABLE 1:** Operational framework used in this review to distinguish mechanistically persuasive HLA associations from clinically actionable signals in infectious-disease pharmacogenetics.

Domain	Relevance for clinical actionability	High-actionability pattern	Main barrier to translation
Phenotype precision	Clinical utility depends on a severe, specific, and reproducibly defined phenotype	Well-defined, clinically or immunologically adjudicated phenotype	Broad syndromic definitions, mixed endpoints, or inconsistent case ascertainment
Replication strength	Actionability requires a robust and transportable association	Large effect size with independent replication	Single-study signal, fragile replication, or strong ancestry dependence
Mechanistic support	Biological plausibility strengthens causal inference and interpretability	Structural, immunopeptidomic, or functional T-cell evidence	Statistical association without clear mechanistic support
Absolute risk and test performance	Relative risk alone is insufficient if predictive value is poor	High NPV and clinically meaningful predictive performance	Low PPV, low event incidence, or incomplete penetrance
Therapeutic or preventive alternatives	Testing is useful only if it can change management	Clear alternative drug, regimen, or preventive strategy	Limited substitution options or unacceptable clinical trade-offs
Feasibility of implementation	A useful marker must be testable at the relevant decision point	Pre-treatment or early-treatment testing feasible within routine care	Delayed results, urgent treatment settings, or limited service availability
Current implementation status	Translational maturity is reflected by integration into practice pathways	Guideline-supported or label-directed use	No formal implementation pathway despite biological plausibility

Legend. This author-defined framework summarizes the criteria used throughout the review to judge whether an HLA association is primarily mechanistically informative or sufficiently decision-relevant to influence clinical practice. Domains should be interpreted in combination rather than isolation.

**TABLE 2:** Mechanistic models proposed for HLA-mediated hypersensitivity to anti-infective drugs, with typical experimental readouts, illustrative examples, key inferences, and limitations. Evidence ratings in the final column are author-defined (A = strongest).

Mechanistic model	Typical experimental readouts	Illustrative anti-infective example(s)	Key inference	Main limitations	Evidence in anti-infectives
Hapten/pro-hapten	Drug–protein adduct MS; T-cell assays; metabolism dependence	Beta-lactams; sulfonamides; flucloxacillin (DILI)	Covalent adducts generate neo-antigens presented by HLA	Not universal; does not explain processing-independent reactions	B
Pharmacological interaction (p-i)	Rapid, processing-independent T-cell activation; fixed-APC assays	Flucloxacillin; beta-lactams (heterogeneous)	Direct, labile drug interaction with HLA and/or TCR can activate T cells	Mechanisms can overlap; hard to quantify *in vivo* contribution	C
Altered peptide repertoire	Immunopeptidomics (LC–MS/MS); crystallography; CD8^+^ assays	Abacavir/HLA-B*57:01	Drug reshapes HLA-bound self-peptides (“altered self”) triggering CD8^+^ responses	Best shown for abacavir; incomplete penetrance remains unexplained	A
TCR repertoire restriction/cross-reactivity	TCR sequencing; clonotype mapping; cross-reactivity experiments	Abacavir/HLA-B*57:01 (selected systems)	Host TCR repertoire may modulate susceptibility among risk-allele carriers	Limited drug-specific evidence beyond abacavir; often inferential	D
Antigen-processing modifiers (ERAP/TAP)	Genetic epistasis; processing assays; comparative immunopeptidomics	Dapsone/HLA-B*13:01 (TAP2 modifiers)	Processing variants may alter peptide supply and modify penetrance	Data limited; LD confounding; not decision-grade yet	D

Legend. Evidence rating is an author-defined, non-formal synthesis reflecting the depth and specificity of mechanistic support in anti-infective drug studies rather than a validated universal grading framework (and not the broader immunology literature): A, strongest convergent support; B, strong support; C, moderate support; D, limited or emerging support. Models are not mutually exclusive; a single drug may engage more than one pathway. Abbreviations: APC, antigen-presenting cell; DILI, drug-induced liver injury; ERAP, endoplasmic reticulum aminopeptidase; HLA, human leukocyte antigen; LC–MS/MS, liquid chromatography–tandem mass spectrometry; MS, mass spectrometry; p-i, pharmacological interaction; TAP, transporter associated with antigen processing; TCR, T-cell receptor.

Incomplete penetrance reflects additional modifiers beyond HLA, including T-cell receptor repertoire, antigen-processing variation, immune context, and drug-exposure determinants (Deshpande et al., 2021; Pavlos et al., 2012; Almeida et al., 2019; Hattori and Tsujimoto, 2013; Reeves and James, 2018).

At one end of the mechanistic spectrum lies the classical hapten/pro-hapten paradigm, in which a drug—or more commonly a reactive metabolite—forms a covalent adduct with endogenous proteins, generating neo-antigens that require processing and HLA-restricted presentation to drug-reactive T cells (Padovan et al., 1997; Naisbitt et al., 2002). Functional human evidence for beta-lactams shows that T cells from allergic patients can recognize penicilloyl-modified self-peptides as antigenic determinants, establishing haptenated peptide–HLA complexes as a biologically plausible basis for T-cell–mediated beta-lactam allergy (Padovan et al., 1997). For sulfonamides, metabolism-dependent formation of reactive intermediates provides a pro-hapten route to covalent binding and MHC-restricted antigen presentation, linking pharmacology (bioactivation) to T-cell immunogenicity in humans (Naisbitt et al., 2002). More recently, mass-spectrometry identification of flucloxacillin-haptenated ligands presented by HLA-B*57:01 provides direct evidence that drug-modified structures can reach the HLA presentation pathway in an anti-infective setting (Waddington et al., 2020).

A conceptually distinct route is captured by the pharmacological interaction (p-i) model, which proposes that some drugs activate T cells through rapid, non-covalent interactions with immune receptors—either HLA (p-i HLA) and/or the T-cell receptor (p-i TCR)—without requiring covalent protein modification or classical antigen processing (Pichler, 2002). This mechanism offers an explanation for hypersensitivity to parent compounds that are not obvious haptens and can rationalize experimental observations of rapid T-cell activation in systems incompatible with processing-dependent presentation (Pichler, 2002). In anti-infective pharmacology, flucloxacillin provides a particularly instructive example because the balance between hapten-type and p-i-type reactivity can be influenced by HLA haplotype context, illustrating that mechanistic routes can be modulated by genetic background rather than being mutually exclusive alternatives (Wuillemin et al., 2013). In beta-lactam allergy more broadly, heterogeneous T-cell responses to drug-modified self-structures underscore the diversity of T-cell recognition pathways that may be engaged across individuals (Brander et al., 1995).

Although most clinically actionable HLA associations concern delayed T-cell-mediated phenotypes, genome-wide and biobank data suggest that HLA may also contribute to immediate beta-lactam hypersensitivity, including an association with HLA-DRB1*10:01 in a deeply phenotyped European cohort and a weaker signal for self-reported penicillin allergy at HLA-B*55:01 in population-scale data (Nicoletti et al., 2021a; Krebs et al., 2020). These immediate reactions should not be assumed to follow the same mechanistic framework as the delayed T cell-mediated HLA-restricted phenotypes discussed below.

The most mechanistically “clean” and clinically consequential anti-infective exemplar is the altered peptide repertoire model, established for abacavir hypersensitivity. Structural and immunopeptidomic studies showed that abacavir binds within the peptide-binding cleft of HLA-B*57:01 and reshapes the repertoire of endogenous peptides presented on the cell surface, effectively generating an “altered-self” landscape recognized by cytotoxic T cells (Illing et al., 2012; Ostrov et al., 2012). Complementary experimental work demonstrated drug-induced loading of novel self-peptides into HLA-B*57:01, supporting an autoimmune-like model of HLA-associated drug hypersensitivity grounded in measurable changes to antigen presentation (Norcross et al., 2012). This mechanistic clarity matters for translation: it provides a molecular explanation for allele specificity and supports a causal narrative linking genotype to drug-dependent antigen presentation.

For clinicians, the value of an HLA association depends less on the odds ratio itself and more on how the test performs in real patients. Because severe immune-mediated ADRs are rare, HLA tests often function primarily as rule-out tools with high negative predictive value, while positive predictive value remains constrained by event incidence and penetrance (Manson et al., 2020; Aithal, 2015). In practical terms, this means that even very large relative effect sizes may still translate into limited PPV when the underlying adverse phenotype is rare. Consistent causality adjudication and phenotype standardization are particularly important for DILI endpoints (Aithal et al., 2011; Danan and Teschke, 2019). Abacavir illustrates an unusually favorable scenario: randomized evidence shows that prospective HLA-B*57:01 screening can prevent immunologically confirmed hypersensitivity, demonstrating actionability when the phenotype is severe, clearly defined, and therapeutic alternatives exist (Mallal et al., 2008). Meta-analytic diagnostic accuracy further underscores a general lesson for this review: phenotype precision matters, with stronger performance when immunologically confirmed endpoints are used (Cargnin et al., 2014; Tangamornsuksan et al., 2015). In subsequent drug-specific sections, we therefore report effect sizes and (when available and verifiable) screening metrics alongside practical implementation considerations, including guideline adoption as a pragmatic marker of readiness (Martin et al., 2012) and health-system constraints that drive real-world feasibility (Hughes et al., 2004; Plumpton et al., 2016; Zhou et al., 2021).

For clarity, the main dimensions that determine whether an HLA association remains mechanistically informative or becomes clinically actionable are summarized in Table 1.

### 2.2 Selected anti-infective drug exemplars

The drug-specific subsections that follow highlight representative HLA-associated adverse drug reactions across major anti-infective classes. These exemplars were selected based on the strength and reproducibility of the genetic signal, availability of mechanistic support, and the extent to which the allele–phenotype relationship has been replicated across independent cohorts. The aim is not to provide an exhaustive catalogue, but to illustrate how phenotype precision, effect size, allele frequency, and the presence of feasible therapeutic alternatives determine whether an HLA association becomes clinically actionable. Key drug–allele pairs and implementation considerations are collated in Table 3 (Manson et al., 2020; Negrini and Becquemont, 2017b). The table includes both clinically mature examples and selected lower-replication or exploratory associations retained to illustrate the translational spectrum of HLA findings across anti-infective pharmacology.

**TABLE 3:** HLA-associated adverse drug reactions to anti-infective drugs: drug–allele pairs, populations, effect estimates, and implementation notes.

Drug	ClinPGx level	HLA allele	ADR	Population	Effect estimate	Implementation	References
Abacavir	1A	B*57:01	Multiorgan HSR	All	DOR 1,141 (IC-HSR); DOR 33.07 (CS-HSR)	Pre-treatment screening recommended/label-directed (FDA/EMA); CPIC provides genotype-based prescribing guidance	Cargnin et al. (2014); Mallal et al. (2008)
Dapsone	2A	B*13:01	DRESS/DHS	Chinese, SE Asians	OR 20.53 (GWAS); pooled OR 43.0	Prospectively implemented in China	Zhang et al. (2013); Tangamornsuksan and Lohitnavy (2018)
Flucloxacillin	1A	B*57:01	Cholestatic DILI	Europeans	OR 80.6	No routine pre-emptive HLA screening recommended; clinical monitoring only	Daly et al. (2009)
Flucloxacillin	N/A	B*57:03	DILI	Europeans	OR 19.77; conditional OR 79.21	Not implemented	Nicoletti et al. (2019)
Amoxicillin–clavulanate	N/A	A*02:01; DRB1*15:01-DQB1*06:02	AC-DILI	Europeans/United States of America	OR 2.2 (A*02:01); OR 3.3 (DQB1*06:02/DRB1*15:01-linked class II signal)	Not implemented	Lucena et al. (2011)
Nevirapine	2B	DRB1*01:01	Hepatic/systemic hypersensitivity (not isolated rash)	Australians (predominantly Caucasian)	OR 4.8; OR 17.7 for HLA-DRB1*01:01 plus CD4 ≥25%	Not routinely recommended	Martin et al. (2005)
Nevirapine	3	B*35:05	Cutaneous rash	Thai, SE Asians	OR 18.96 (95% CI 4.87–73.44)	Population-specific association reported; not routinely implemented	Chantarangsu et al. (2009)
Nevirapine	3	B*58:01; DRB1*01:02	Hepatotoxicity	South Africans	RR 3.81 (B*58:01); RR 4.72 (DRB1*01:02), multivariable	Not implemented	Phillips et al. (2013)
Efavirenz	N/A	DRB1*01:01	Cutaneous hypersensitivity	Europeans (French)	P = 0.004 (pc = 0.04); OR not formally calculated (n = 21)†	Not implemented	Vitezica et al. (2008)
Cotrimoxazole	N/A	B*15:02/C*08:01 haplotypes	SJS/TEN	Thai, SE Asians	Haplotype OR 14.75–18.26	Emerging	Kongpan et al. (2015)
Cotrimoxazole	N/A	B*13:01	DRESS	East/Southeast Asian; meta-analysis	OR 23.09 (DRESS subgroup); OR 5.96 (overall SCAR vs. tolerant controls)	Emerging	Wu et al. (2024)
Cotrimoxazole	N/A	B*38:02	SJS/TEN	East/Southeast Asian; meta-analysis	OR 5.13 (SJS/TEN subgroup); OR 3.47 (overall SCAR vs. tolerant controls)	Not implemented	Wang et al. (2021); Wu et al. (2024)
Cotrimoxazole	N/A	B*44:03; B*38:01; C*04:01	SJS/TEN, DRESS	United States of America, South Africa	US SJS/TEN: OR 4.68 (B*44:03), 4.33 (B*38:01), 2.95 (C*04:01); South African DRESS: OR 10.69 (B*44:03)	Not implemented	Li et al. (2025)
Isoniazid (HLA)	N/A	B*52:01	Hepatocellular DILI	Indians + Europeans	OR 2.67	Not implemented	Nicoletti et al. (2021b)
Rifampicin	N/A	B*57:03; B*57:02 (serogroup B*57)	DILI (TB + ARV co-treatment)	Ethiopians	Carrier proportion 37.0% vs. 2.2% (cases vs. tolerant controls); allele freq 25% vs. 1.1%	Not implemented	Petros et al. (2017)
Vancomycin	3	A*32:01	DRESS	Europeans, Americans	19/23 cases vs. 0/46 matched controls; 19.2% among exposed carriers developed DRESS	Not implemented; may assist culprit attribution in complex cases	Konvinse et al. (2019)
Minocycline	3	B*35:02	Autoimmune DILI (ANA+)	Americans (DILIN)	OR 29.6	Not implemented	Urban et al. (2017)
Nitrofurantoin	N/A	DRB1*11:04	Autoimmune DILI	United States of America (DILIN)	OR 4.29 vs. population controls (95% CI 2.18–7.66)	Not implemented	Chalasani et al. (2023)
Terbinafine	N/A	A*33:01	Cholestatic DILI	Predominantly European ancestry (DILIN)	10/11 European-ancestry cases (91%) vs. 1.6% population controls	Not implemented	Fontana et al. (2018)
Fluoroquinolones	N/A	DQA1*03:01; B*57:01	Mixed DILI	United States of America (DILIN)	Carriage: DQA1*03:01 38% vs. 19%; B*57:01 15% vs. 6%; combined 48% vs. 24% (P = 0.0001)	Not implemented	Ahmad et al. (2026)
Levofloxacin	N/A	B*13:01; B*13:02 (B13 serotype)	SCAR (SJS/TEN, DRESS)	Han Chinese	B*13:01 OR 4.50; B*13:02 OR 6.14; B13 serotype OR 17.73	Not implemented	Jiang et al. (2023)
Penicillins (general)	3	B*55:01	Self-reported allergy	Multiethnic (>1M)	OR 1.41; replication OR 1.30	Not implemented	Krebs et al. (2020)
Beta-lactams	N/A	DRB1*10:01	Immediate HSR (IgE)	Europeans	OR 2.93	Not implemented	Nicoletti et al. (2021a)
Metronidazole	N/A	A*24:02	Cutaneous reactions	Han Chinese	OR 5.80 vs. population controls; OR 7.56 vs. tolerant controls	Not implemented	Yang et al. (2020)
Voriconazole	N/A	B*35:02	DILI; photosensitivity	Case report	Not quantifiable (single case report)§	Not implemented	Bühler et al. (2019)

Legend. ClinPGx level refers to ClinPGx (formerly PharmGKB) Summary Annotation Levels of Evidence (1A-4). Assignments reflect the exact association when available or the closest matching gene/drug/phenotype summary annotation when an exact match was unavailable. N/A indicates that no ClinPGx Summary Annotation was identified for the exact drug-HLA association or allele combination shown in the row. ClinPGx evidence level should not be interpreted as equivalent to guideline endorsement or routine clinical implementation. Abbreviations: ADR, adverse drug reaction; ANA, antinuclear antibodies; ARV, antiretroviral; CS-HSR, clinically suspected hypersensitivity reaction; DHS, dapsone hypersensitivity syndrome; DILI, drug-induced liver injury; DOR, diagnostic odds ratio; DRESS, drug reaction with eosinophilia and systemic symptoms; HLA, human leukocyte antigen; HSR, hypersensitivity reaction; IC-HSR, immunologically confirmed hypersensitivity reaction; OR, odds ratio; RR, relative risk; SCAR, severe cutaneous adverse reaction; SJS/TEN, Stevens-Johnson syndrome/toxic epidermal necrolysis; TB, tuberculosis. The lower portion of the table also includes exploratory or lower-replication HLA associations that are not all individually discussed in the main text; they are retained for reference. Rows supported by prospective screening, implementation studies, randomized evidence, or formal guideline/regulatory integration should be interpreted as translationally more mature than associations retained primarily for mechanistic, epidemiological, or reference purposes. For some associations, study-specific relative risks or descriptive case/control frequencies are reported when these were the primary effect measures in the source study and are not directly comparable with pooled odds ratios cited elsewhere in the text. † Exploratory finding from a very small cohort (n = 21); OR not formally calculated in the source. § Single case report; included for completeness only. Evidence insufficient for any inferential conclusion. ClinPGx, definitions follow (Whirl-Carrillo et al., 2021).

#### 2.2.1 Abacavir

Abacavir is a nucleoside analogue reverse transcriptase inhibitor used in combination antiretroviral regimens for HIV-1 infection (FDA, 2020). Its major safety liability is abacavir hypersensitivity reaction (ABC-HSR), a distinctive, immune-mediated multisystem syndrome that can progress with continued dosing and may be fatal if abacavir is restarted after a suspected reaction (Chaponda and Pirmohamed, 2011; FDA, 2020). Clinical diagnostic criteria have been described to require at least two symptoms among fever, rash, nausea, vomiting, headache, lethargy, myalgia, arthralgia, or gastrointestinal symptoms, typically occurring within the first 6 weeks of therapy and resolving within 72 h of withdrawal of the drug; less frequent manifestations include respiratory symptoms, paresthesia, edema, and renal or hepatic failure (Chaponda and Pirmohamed, 2011). Regulatory labeling also provides an operational “symptom-group” framework: hypersensitivity is usually characterized by a sign or symptom in two or more of five symptom groups—(1) fever, (2) rash, (3) gastrointestinal symptoms (including nausea, vomiting, diarrhea, or abdominal pain), (4) constitutional symptoms (including generalized malaise, fatigue, or achiness), and (5) respiratory symptoms (including dyspnea, cough, or pharyngitis)—and abacavir should be discontinued as soon as hypersensitivity is suspected (FDA, 2020). If hypersensitivity cannot be ruled out, abacavir should not be restarted, because more severe symptoms (including life-threatening hypotension and death) can occur within hours (FDA, 2020).

The association between ABC-HSR and HLA-B*57:01 was first reported in 2002 by independent groups, establishing an immunogenetic basis for what had previously been recognized clinically during early development and post-marketing use (Mallal et al., 2002; Hetherington et al., 2002). Definitive prospective evidence came from PREDICT-1, a double-blind randomized trial (n = 1,956; 19 countries) designed to test whether pre-treatment HLA-B*57:01 screening could prevent ABC-HSR (Mallal et al., 2008). In PREDICT-1, the prevalence of HLA-B*57:01 was 5.6% (109/1,956), and among abacavir recipients overall, 84% were White and 72% were men (Mallal et al., 2008). Screening eliminated immunologically confirmed ABC-HSR (0% in the prospective-screening group vs. 2.7% in the control group) and reduced clinically suspected reactions (3.4% vs. 7.8%), with a negative predictive value (NPV) of 100% and a positive predictive value (PPV) of 47.9% for immunologically confirmed ABC-HSR (Mallal et al., 2008). Concerns about transferability beyond predominantly White trial populations were subsequently addressed by SHAPE, which used patch-test-defined cases and demonstrated 100% sensitivity of HLA-B*57:01 for immunologically confirmed ABC-HSR in both White and Black participants; sensitivity was substantially lower when case definitions relied on clinical diagnosis alone (Saag et al., 2008). Early prospective cohort screening in Western Australia (n = 260) reported no ABC-HSR among 148 HLA-B*57:01-negative abacavir recipients, providing early real-world confirmatory evidence (Rauch et al., 2006).

Mechanistically, ABC-HSR is the paradigmatic example of the altered peptide repertoire model of HLA-associated, T-cell–mediated drug hypersensitivity. Structural and immunopeptidomic studies demonstrated that abacavir binds non-covalently within the peptide-binding cleft of HLA-B*57:01, altering the repertoire of self-peptides presented and triggering CD8^+^ T-cell responses against these “altered-self” complexes (Illing et al., 2012; Ostrov et al., 2012). Norcross et al. further supported this model by showing abacavir-dependent loading and detection of novel self-peptides on HLA-B*57:01 (Norcross et al., 2012). Consistent with an HLA class I–restricted mechanism, systemic drug hypersensitivity in this setting is mediated by HLA-restricted activation of CD8^+^ T cells (Chessman et al., 2008).

Allele-level specificity matters clinically: abacavir-responsive T-cell activation is restricted to HLA-B*57:01, whereas closely related HLA-B*57:02 and HLA-B*57:03 do not reproduce the same response; conversely, HLA-B*57:02/*57:03, rather than HLA-B*57:01, have been linked to liver toxicity during anti-tuberculosis plus antiretroviral co-treatment, underscoring the need for high-resolution HLA typing when interpreting drug-safety associations (Chessman et al., 2008; Illing et al., 2012; Petros et al., 2017).

Implementation has been formalized in major guidelines and drug labelling. CPIC recommends HLA-B*57:01 testing before initiating abacavir and advises that HLA-B*57:01-positive individuals should not receive abacavir; the 2014 CPIC update maintained these recommendations (Martin et al., 2012; Martin et al., 2014). U.S. prescribing information similarly recommends pre-treatment screening, prompt discontinuation when hypersensitivity is suspected, and permanent avoidance of abacavir rechallenge when hypersensitivity cannot be ruled out (FDA, 2020). Health-economic evaluations have supported screening in settings where abacavir is used, driven by the preventable morbidity of ABC-HSR and the availability of effective alternatives within antiretroviral regimens (Hughes et al., 2004; Nolan, 2009). A recurring pattern in HLA-associated hypersensitivity is that the risk allele is often necessary but not sufficient, yielding a clinically valuable exclusion test even when positive predictive value remains modest (Negrini and Becquemont, 2017a; Negrini and Becquemont, 2017b).

#### 2.2.2 Dapsone

Dapsone remains central to multidrug therapy for leprosy and is also used in several inflammatory dermatoses. Its most feared immune-mediated toxicity is dapsone hypersensitivity syndrome (DHS), a systemic idiosyncratic reaction that clinically overlaps with DRESS/DIHS and typically occurs within a few weeks of starting therapy, with common features including fever, rash, hepatitis, lymphadenopathy, and eosinophilia; historical series suggest an incidence in the range of 0.5%–3.6% of exposed patients and a ∼10% fatality rate (Zhang et al., 2013; Wang et al., 2017). The pivotal genetic discovery came from a landmark GWAS in Chinese leprosy patients receiving multidrug therapy, which identified HLA-B*13:01 as the dominant risk allele for DHS (OR 20.53, p = 6.84 × 10^−25^) with reported sensitivity 85.5% and specificity 85.7%; absence of HLA-B*13:01 was associated with a marked reduction in DHS risk (from 1.4% to 0.2%) (Zhang et al., 2013). Subsequent studies broadened and refined the phenotype to dapsone-induced severe cutaneous adverse reactions (SCAR), encompassing predominantly DRESS but also SJS/TEN (Stevens-Johnson Syndrome and Toxic Epidermal Necrolysis) in some cohorts, supporting cross-population reproducibility in Asian settings (Tempark et al., 2017).

A systematic review and meta-analysis further quantified the association: pooled estimates showed a very strong relationship between HLA-B*13:01 and dapsone-induced cutaneous adverse drug reactions overall (pooled OR 43.0, 95% CI 24.0–77.2), with similarly high effect estimates reported in subgroup analyses for DHS and other severe phenotypes (Tangamornsuksan and Lohitnavy, 2018). Importantly, translation to prevention has been demonstrated prospectively. In a large implementation study across multiple Chinese provinces (n = 1,512), pre-prescription HLA-B*13:01 screening was used to withhold dapsone from carriers; no DHS cases occurred in the screened cohort compared with a historical DHS incidence of ∼1.0% (Liu et al., 2019). Although penetrance is incomplete and the PPV is modest—with tolerance mechanisms and additional host factors likely contributing—available data support a high NPV in screened populations, making the genotype clinically useful as a “rule-out” tool where the allele is prevalent and alternative regimens exist (Liu et al., 2019; Sun et al., 2023). Mechanistic work also supports biological plausibility: *in vitro* studies show HLA-B*13:01-dependent cytotoxic T-cell activation by dapsone; more broadly, granulysin-mediated effector injury is well established in SCAR, consistent with a T-cell-mediated reaction pathway (Chen et al., 2018; Chung et al., 2008). Taken together, the dapsone–HLA-B*13:01 paradigm illustrates that screening can be justified even with moderate PPV when the reaction is severe, potentially fatal, and preventable through substitution strategies in high-prevalence settings (Zhang et al., 2013; Liu et al., 2019).

#### 2.2.3 Flucloxacillin

Flucloxacillin is a well-established cause of cholestatic liver injury with delayed onset, complicating causal attribution in routine care (Olsson et al., 1992; Russmann et al., 2005).

A pivotal genome-wide association study first identified a major susceptibility signal in the MHC region in strong linkage disequilibrium with HLA-B*57:01, demonstrating a very large effect size for flucloxacillin DILI (OR 80.6) (Daly et al., 2009). A subsequent larger GWAS (197 cases, 6,835 controls) consolidated the central role of HLA-B*57:01 (allelic OR 36.62, P = 2.67 × 10^−97^) and also identified an additional signal at HLA-B*57:03 (OR 19.77 in the primary analysis; conditional OR 79.21 in reciprocal conditional analyses), with fine-mapping implicating an HLA-B amino acid residue (valine at position 97) shared by the risk alleles (Nicoletti et al., 2019). Importantly, despite high relative risks, the absolute event rate remains very low (on the order of ∼0.01%), which constrains the clinical utility of routine population screening for this drug alone (Nicoletti et al., 2019; Russmann et al., 2005).

*In vitro* work using cells from affected patients and HLA-matched donors has demonstrated HLA-B*57:01-restricted flucloxacillin-responsive T-cell activation, providing biological plausibility for drug-specific antigen presentation and cytotoxic effector responses in susceptible individuals (Monshi et al., 2013). Nevertheless, unlike the abacavir/HLA-B*57:01 paradigm, flucloxacillin is not currently included in CPIC or DPWG prescribing recommendations for pre-emptive testing, reflecting the combination of low incidence and modest real-world preventable yield (Manson et al., 2020). Even when pre-emptive screening is not justified, HLA testing may still retain value as a diagnostic or exclusion tool in suspected cases, particularly when differential attribution remains clinically important (Negrini and Becquemont, 2017a; Negrini and Becquemont, 2017b). In practice, the main translational value of the association is therefore conceptual: it exemplifies how a strong HLA signal can coexist with low positive predictive value when the underlying adverse event is rare, and it motivates careful phenotyping and pharmacovigilance rather than routine pre-prescription exclusion (Russmann et al., 2005; Manson et al., 2020).

#### 2.2.4 Vancomycin

Vancomycin is a glycopeptide antibiotic widely used for severe Gram-positive infections, but it is also a well-recognized trigger of drug reaction with eosinophilia and systemic symptoms (DRESS), a delayed, T cell–mediated, multi-organ hypersensitivity syndrome that typically develops after several weeks of exposure and may be complicated by hepatic involvement (Pirmohamed, 2019; Kardaun et al., 2013; Shiohara and Mizukawa, 2019; Hama et al., 2022). This entity is distinct from the vancomycin infusion reaction (formerly “red man syndrome”), which is an infusion-rate–dependent histamine release phenomenon and is not HLA-associated (Pirmohamed, 2019; Kardaun et al., 2013; Shiohara and Mizukawa, 2019; Hama et al., 2022).

The genetic basis of vancomycin-induced DRESS was established by Konvinse and colleagues, who showed marked enrichment of HLA-A*32:01 among adjudicated cases, with 19/23 cases (82.6%) carrying the allele versus 0/46 matched vancomycin-tolerant controls and 6.3% of the BioVU population (Konvinse et al., 2019). Time-to-event analysis further suggested that 19.2% of exposed carriers developed DRESS within 4 weeks, indicating a strong association but incomplete penetrance (Konvinse et al., 2019). Replication and extension across settings have been reported, including liver-injury phenotypes and East Asian cohorts, with independent studies generally supporting a substantial but not absolute effect size (Asif et al., 2024; Wang et al., 2022; Krantz et al., 2023). Mechanistic evidence remains preliminary, but molecular docking analyses have suggested a plausible interaction between vancomycin and HLA-A*32:01, supporting biological plausibility.

Despite consistent genetic association signals, there is currently no CPIC/DPWG or regulatory recommendation for routine pre-prescription HLA-A*32:01 screening before vancomycin. At present, HLA typing is best viewed as an adjunct that may support diagnosis and culprit assignment in complex clinical scenarios, and as a foundation for future work on risk stratification in patients anticipated to require prolonged vancomycin courses (Gardner et al., 2025).

#### 2.2.5 Nevirapine

In a Western Australian cohort, Martin et al. reported that susceptibility to nevirapine hepatic/systemic hypersensitivity was associated with HLA-DRB1*01:01 and was strongly modified by baseline immune status, with higher CD4^+^ T-cell percentages amplifying risk, whereas isolated cutaneous rash showed no clear association in that study (Martin et al., 2005). For cutaneous reactions in Thai patients, Chantarangsu et al. identified HLA-B*35:05 as a strong predictor of nevirapine-induced skin adverse drug reactions, with a large effect size (OR 18.96, 95% CI 4.87–73.44) (Chantarangsu et al., 2009). A systematic review and meta-analysis integrating multi-ethnic studies confirmed that different alleles track with different phenotypes: HLA-C*04 was the most consistent cross-ethnic signal for cutaneous adverse drug reactions (pooled OR 2.63, 95% CI 1.97–3.52; I^2^ = 0%), while HLA-DRB1*01 was most strongly associated with hepatotoxicity (pooled OR 2.94, 95% CI 1.92–4.50; I^2^ = 0%), and HLA-B*58:01 conferred hepatotoxicity risk in Black African populations (pooled OR 3.51, 95% CI 1.72–7.19; I^2^ = 0%) (Cornejo Castro et al., 2015). Mechanistically, Pavlos et al. supported a structure-based explanation for phenotype specificity, showing that cutaneous hypersensitivity clustered with shared HLA peptide-binding features centred on HLA-C*04:01 (Pavlos et al., 2017). Genome-wide data from sub-Saharan Africa further support phenotype- and ancestry-dependent architecture (Carr et al., 2017).

Despite replicated associations, no routine pre-prescription HLA screening strategy has been adopted for nevirapine because risk alleles differ across ancestries and phenotypes and predictive values remain limited at the point of care (Cornejo Castro et al., 2015). Consequently, clinical risk mitigation continues to rely primarily on label-based risk stratification and intensive early monitoring, including avoidance of initiation in women with CD4^+^ >250 cells/mm^3^ and in men with CD4^+^ >400 cells/mm^3^ unless the benefit outweighs the risk (FDA, 2024; European Medicines Agency, 2024).

#### 2.2.6 Amoxicillin–clavulanate

Amoxicillin–clavulanate is a leading cause of idiosyncratic DILI in Western registries, typically presenting as cholestatic or mixed hepatitis with jaundice and pruritus after a latency of several weeks; onset may occur after the antibiotic course has been completed (deLemos et al., 2016; Lucena et al., 2011). Early HLA work showed a strong enrichment of the class II haplotype HLA-DRB1*15:01–DRB5*01:01–DQB1*06:02 in biopsy-confirmed cases (57.1% of cases vs. 11.7% of controls; corrected P < 0.0002), supporting an immune-mediated mechanism (Hautekeete et al., 1999). A larger United Kingdom multicentre study confirmed HLA-DRB1*15 as a susceptibility factor (OR 2.59; 95% CI 1.44–4.68) and identified HLA-DRB1*07 as potentially protective (OR 0.27; 95% CI 0.11–0.65), highlighting that class II variation can influence both risk and resistance (Donaldson et al., 2010).

Mechanistic studies support adaptive immunity: both amoxicillin- and clavulanate-responsive T cells have been demonstrated in affected patients, and HLA-DRB1*15:01–HLA-DQB1*06:02–restricted CD4^+^ T cells can be selectively activated by amoxicillin–peptide adducts (Kim et al., 2015; Tailor et al., 2020). Despite robust associations, routine pre-prescription HLA screening is not currently implemented, largely because the absolute incidence of this reaction is low and predictive values for population-wide testing remain unfavorable (Nicoletti et al., 2022).

#### 2.2.7 Nitrofurantoin

Nitrofurantoin is a recognized cause of idiosyncratic DILI, particularly in the setting of prolonged prophylactic use for recurrent urinary tract infections. In the largest clinical–genetic series from the U.S. Drug-Induced Liver Injury Network (DILIN), 78 adjudicated nitrofurantoin-DILI cases showed a predominantly hepatocellular pattern (69%) and were frequently icteric (55%). Distinct exposure profiles were observed (≤7 days, 8–364 days, and ≥1 year), with long-exposure cases (44/78) displaying a more autoimmune-like signature, including higher rates of AST>ALT (73%) and ANA/SMA positivity (91%) compared with shorter exposures, and more frequent corticosteroid use. Clinically important outcomes occurred, including death and liver transplantation (Chalasani et al., 2023). HLA typing in the DILIN cohort identified HLA-DRB1*11:04 as a susceptibility allele (OR 4.29 vs. population controls) (Chalasani et al., 2023), while an independent European cohort reported a distinct signal at HLA-A*33:01 (OR 10.93) (Daly et al., 2023).

Mechanistic evidence remains limited, but immune-mediated injury is biologically plausible and has been supported by early histological observations consistent with CD8^+^ T-cell involvement (Kelly et al., 1998). Overall, nitrofurantoin-DILI illustrates the challenge of translating HLA signals to screening when replication across populations is inconsistent and absolute event rates are low (Daly et al., 2023).

#### 2.2.8 Anti-tuberculosis drugs

Antituberculosis drug-induced liver injury (AT-DILI) is an idiosyncratic adverse reaction that most often presents as hepatocellular injury during standard first-line therapy (isoniazid, rifampicin, pyrazinamide ± ethambutol). Clinically, AT-DILI is commonly defined by alanine aminotransferase (ALT) elevations >3× the upper limit of normal (ULN) with symptoms or >5× ULN without symptoms, typically emerging within the first 2–8 weeks of treatment initiation, although incidence varies widely across studies and case definitions (Cai et al., 2012). While metabolic susceptibility—particularly variability in drug-metabolising enzymes affecting isoniazid handling—is the best-established pharmacogenetic component, a smaller and less consistent body of evidence suggests that HLA-restricted adaptive immune mechanisms may contribute in a subset of patients (Nicoletti et al., 2021b; Bao et al., 2018).

The currently reported HLA signal in antituberculosis drug-induced liver injury remains suggestive rather than decision-grade. In a trans-ethnic genome-wide study of DILI due to isoniazid-containing regimens, HLA-B*52:01 showed a modest association with AT-DILI (OR 2.67, 95% CI 1.63–4.37; P = 9.4 × 10^−5^), with an additional European-specific signal at rs117491755 (Nicoletti et al., 2021b). These data support a possible HLA contribution, but the signal did not reach the evidentiary maturity of high-penetrance HLA–ADR pairs, independent genome-wide replication is lacking, and current clinical pharmacogenetic relevance remains dominated by non-HLA factors such as NAT2.

Candidate-gene studies of HLA class II loci have also reported inconsistent results across populations and case definitions, underscoring limited transportability at present; in addition, the clinical translation of even moderate HLA signals is intrinsically difficult in this setting because first-line anti-tuberculosis therapy is delivered as a multidrug regimen and therapeutic substitution pathways are less straightforward than in most other anti-infective hypersensitivity scenarios (Chen et al., 2015; Leiro-Fernández et al., 2016; Prayuni et al., 2023).

#### 2.2.9 Other anti-infective drugs

Several additional anti-infectives have been linked to HLA-associated idiosyncratic toxicity, but the evidence base is smaller and clinical translation remains limited. Co-trimoxazole is a notable example because HLA signals have now been reported across severe cutaneous phenotypes, including SJS/TEN and DRESS. In Southeast Asian cohorts, HLA-B*15:02 together with HLA-C*08:01 was associated with co-trimoxazole-induced SJS/TEN, while more recent studies identified HLA-B*13:01 and shared HLA-B peptide-binding motifs as risk markers for co-trimoxazole-related SCAR, including DRESS, across multiple populations (Kongpan et al., 2015; Wu et al., 2024; Li et al., 2025). However, phenotype heterogeneity, ancestry dependence, and the absence of implementable prospective performance data currently preclude routine pre-emptive screening. Other reported examples include terbinafine-related liver injury with enrichment signals involving HLA-A*33:01 (Fontana et al., 2018; Nicoletti et al., 2017), minocycline-associated autoimmune-like hepatitis with a high-effect HLA class I signal (Urban et al., 2017; Czaja, 2011), and emerging HLA associations for cephalosporin-related phenotypes in specific populations (Vuppalanchi et al., 2025; Wang et al., 2024; Wattanachai et al., 2023; Nakkam et al., 2018). At present, neither CPIC nor DPWG recommends pre-emptive HLA screening for these agents.

### 2.3 Clinical implementation and limitations of HLA screening for anti-infectives

Whether pre-prescription HLA testing is clinically actionable depends on several converging conditions rather than on effect size alone: the prevented phenotype must be severe and reproducibly defined, the test must show useful real-world performance, a clinically acceptable alternative must exist, and the population context must make screening meaningful in terms of prevalence, feasibility, and net benefit. From a translational perspective, the most credible HLA-guided interventions are those in which phenotype severity, high negative predictive value, actionable prescribing alternatives, and a realistic testing burden converge (Negrini and Becquemont, 2017a; Negrini and Becquemont, 2017b). Abacavir/HLA-B*57:01 remains the clearest implementation paradigm. Randomized prospective evidence demonstrates that pre-treatment HLA-B*57:01 screening can prevent immunologically confirmed hypersensitivity (Section 2.2.1) (Mallal et al., 2008), and meta-analytic data confirm higher diagnostic performance when outcomes are immunologically confirmed (Cargnin et al., 2014). Regulatory positioning aligns with implementation: the U.S. prescribing information and the EMA SmPC recommend (and operationally require) screening prior to use; CPIC provides genotype-based prescribing recommendations for abacavir when an HLA-B*57:01 result is available, while the practical “requirement” to screen is driven by regulatory labeling (FDA, 2020; EMA, 2026; Martin et al., 2012; Martin et al., 2014). Cost-effectiveness analyses have reported favorable ICERs and, in some settings, dominant (cost-saving) results, although estimates are context-dependent (Hughes et al., 2004; Schackman et al., 2008). Evidence frameworks for pharmacogenomic implementation emphasize the same decision-oriented requirements (Whirl-Carrillo et al., 2021). In other words, clinical testing is justified only when biological association, phenotype severity, predictive performance, management consequences, and population-level feasibility converge sufficiently to change real-world decisions.

Not all strong associations translate into routine screening. Dapsone hypersensitivity syndrome (DHS) is severe and carries substantial mortality, and HLA-B*13:01 confers a strong risk (Zhang et al., 2013; Tangamornsuksan and Lohitnavy, 2018). Prospective screening data support feasibility and high NPV (Liu et al., 2019). However, PPV remains constrained (reported at 7.8%; Sun et al., 2023) because DHS is uncommon, so many carriers would be denied dapsone despite never developing DHS—an acceptable trade-off mainly in high-prevalence settings where substitution is feasible within local practice. This limitation becomes clinically decisive when the “alternative” is limited or unsafe. In a Thai HIV setting, HLA-B*13:01 screening before co-trimoxazole was reported as dominated (lower QALYs with higher cost) when the downstream consequence for carriers was loss of effective Pneumocystis prophylaxis (Kategeaw et al., 2023).

Flucloxacillin DILI highlights the same absolute-risk problem. Although HLA-B*57:01 confers a very high odds ratio (OR 80.6), the incidence of DILI is low, collapsing PPV and inflating the reported NNG to a level that makes universal screening clinically and economically unattractive (Daly et al., 2009). Consistent with this, routine screening has not been adopted in guidelines or labeling (Nicoletti et al., 2019; Manson et al., 2020). Vancomycin DRESS associated with HLA-A*32:01 is an emerging signal with strong case-control enrichment, but prospective validation and implementable performance metrics remain insufficient for routine screening (Konvinse et al., 2019; Krantz et al., 2023).

## 3 HLA-region associations with infection outcomes and vaccine immunogenicity

### 3.1 Immunological mechanisms relevant to infection outcomes

The adaptive immune response to infection depends on the ability of human leukocyte antigen (HLA) molecules to present pathogen-derived peptide fragments to T lymphocytes, and the two classes of HLA molecules do so through distinct intracellular pathways. HLA class I molecules (HLA-A, -B, -C), expressed on virtually all nucleated cells, sample peptides generated from intracellular proteins: these are degraded by the proteasome in the cytosol, transported into the endoplasmic reticulum by the TAP (transporter associated with antigen processing) complex, and loaded onto nascent class I molecules for surface display (Neefjes et al., 2011; Rock et al., 2016). The resulting peptides are short, typically 8 to 10 amino acid residues and are recognized by CD8^+^ cytotoxic T lymphocytes, which kill infected cells directly. HLA class II molecules (HLA-DR, -DQ, -DP), expressed primarily on professional antigen-presenting cells such as dendritic cells and macrophages, follow a different route: extracellular antigens are internalized by endocytosis, degraded by cathepsins in acidified endosomal compartments, and the resulting longer peptides are loaded onto class II molecules with the assistance of HLA-DM, which edits the peptide cargo for stable binding (Neefjes et al., 2011; Kelly and Trowsdale, 2019; Murphy and Weaver, 2017; Busch et al., 2005). These peptide–class II complexes are recognized by CD4^+^ helper T cells, which orchestrate both humoral immunity—by providing cognate help to B cells for antibody class switching and affinity maturation—and cellular responses, including support for CD8^+^ T cell expansion and memory (Sant et al., 2007).

Cross-presentation by dendritic cells can also route exogenous antigens into class I presentation, further connecting antigen handling to CD8^+^ priming (Joffre et al., 2012).

Each HLA allele possesses a peptide-binding groove whose shape is dictated by polymorphic residues. The “binding motif” of an allele describes the specific pattern of amino acids—particularly at anchor positions—that a peptide must carry to fit stably into that groove. The “peptide repertoire” is the full set of peptides an allele can present, and an “immunodominant epitope” is a peptide that elicits a disproportionately strong T-cell response relative to others derived from the same pathogen. Immunodominance is heavily shaped by peptide–HLA binding affinity: Yewdell and Bennink (1999) estimated that only approximately 1 in 200 potential determinants binds a given class I allele at or above the threshold affinity associated with immunogenicity (Kd ≤ 500 nM, i.e., binding affinity of 500 nM or stronger), and T-cell repertoire constraints further halve this number. In HIV infection, HLA class I–peptide stability strongly predicts CD8^+^ immunodominance hierarchies, with alleles associated with viral control preferentially stabilized by epitopes from the abundant Gag protein (Kaseke et al., 2021). For CD4^+^ responses, immunodominance in influenza is governed by similar principles of antigen processing and peptide–class II stability, which focus the helper response onto a limited set of viral epitopes critical for coordinating downstream immunity (Sant et al., 2007). At a translational level, several concepts require clear distinction: relative association strength, absolute risk, phenotype definition, ancestry-dependent transportability, and the possibility that MHC-region linkage disequilibrium may tag broader haplotypic effects rather than a single causal allele. For a pharmacologist evaluating whether an HLA–infection association is “actionable,” several criteria apply. Effect sizes should be reported in both absolute (risk difference) and relative (odds or hazard ratio) terms, because a large relative risk in a rare genotype may have negligible population impact. Replication across independent cohorts and ancestries is essential, given that HLA allele frequencies vary dramatically among populations (Hill, 2001; Attia et al., 2009). The phenotype must be precisely defined — “HIV infection” versus “viral-load set-point” versus “progression to AIDS” engage different immunological mechanisms—and mechanistic plausibility, such as demonstration that the associated allele presents a known immunodominant epitope, strengthens causal inference. Common threats to validity include population stratification, exposure heterogeneity, endpoint heterogeneity, treatment-era confounding, and multiple testing (Attia et al., 2009). For multi-strain pathogens, an allele’s apparent protectiveness may additionally depend on its population frequency through frequency-dependent selection (White et al., 2020; Lipsitch et al., 2003).

### 3.2 Selected infection outcomes and vaccine responses

The examples of infection outcomes and vaccine responses presented below were chosen because they represent some of the most replicated and mechanistically coherent HLA associations in the infectious-disease literature. These cases illustrate how allelic variation in antigen presentation can influence viral control, spontaneous clearance, or vaccine immunogenicity, while also highlighting the methodological and biological factors—heterogeneous endpoints, ancestry-related allele distribution, treatment-era effects, and differing typing resolution—that often limit transportability across settings (Fellay et al., 2007; McLaren et al., 2015; Heim et al., 2016; Li et al., 2013).

#### 3.2.1 HIV-1

Of all infectious diseases studied to date, HIV-1 provides the deepest evidence base for HLA-mediated effects on clinical outcomes, with associations that have survived 2 decades of replication across cohorts and ancestries. The most robust signals involve HLA class I alleles, which present viral peptides to CD8^+^ cytotoxic T lymphocytes and thereby shape the capacity for early viral containment (Fellay et al., 2007; International HIV Controllers Study, 2010; McLaren et al., 2015).

The strongest protective signal belongs to the HLA-B*57 group. In a European cohort, the landmark Euro-CHAVI genome-wide association study identified rs2395029 in HCP5, a single-nucleotide polymorphism tagging HLA-B*57:01 in that dataset, which explained 9.6% of the variation in viral load during the asymptomatic set-point period of infection; together with an independent HLA-C signal, the major MHC associations accounted for 14.1% of set-point variability (Fellay et al., 2007). This finding was confirmed across ancestry in a whole-genome study of 515 African Americans, where HLA-B*57:03 reached genome-wide significance for viral-load set point (P = 5.6 × 10^-10) (Pelak et al., 2010). The International HIV Controllers Study—one of the largest genetic studies of HIV control to date, comparing controllers with progressors of European, African-American, and Hispanic descent—reported that genome-wide significant variants mapped to the MHC region, and that fine-mapping at the amino acid level implicated residues within classical HLA class I molecules consistent with a peptide-presentation mechanism (International HIV Controllers Study, 2010). More recently, re-analysis in the International Collaboration for the Genomics of HIV identified a 1.9-megabase haploblock tagging HLA-B*57:01, comprising 379 SNP alleles impacting 72 genes, raising the possibility that linkage to broader MHC variation could contribute to the magnitude of the B57 effect beyond classical antigen presentation alone (Rahmouni et al., 2023). Micropolymorphism within the B57 group also matters: in >2,000 C-clade infections from southern Africa, HLA-B*57:03 was associated with a median viral-load set point of 5,980 copies/mL compared with 15,190 for HLA-B*57:02 and 19,000 for HLA-B*58:01 (P = 0.24 and P = 0.0005, respectively) (Kloverpris et al., 2012). In an analysis of HIV controllers, HLA-B*57 was independently associated with the long-term non-progressor controller phenotype (OR = 1.924, 95% CI: 1.252–2.957, P = 0.003 in an international validation cohort of 914 controllers) (Dominguez-Molina et al., 2017).

HLA-B*27 is a second replicated protective allele and is commonly linked to control through presentation of conserved Gag epitopes and escape pathways with fitness costs (Hendel et al., 1999; Limou and Zagury, 2013).

Beyond classical HLA-B effects, regulatory variation linked to HLA-C expression may also modulate HIV control, consistent with evidence that higher surface HLA-C expression associates with lower viral load and more favorable disease trajectories in some cohorts (Corrah et al., 2011; Kulkarni et al., 2011).

The best-characterized risk signal is HLA-B*35, particularly its Px subset, which is associated with faster progression in classic seroconverter cohorts (Gao et al., 2001). Proposed mechanisms may include altered interactions with inhibitory receptors on dendritic cells (Huang et al., 2009).

A recurring theme is that protective HLA effects are shaped not only by which epitopes are targeted, but by whether escape is forced into high-cost pathways and whether the responding clonotypes retain functional sensitivity and cross-recognition of early variants (Gorin et al., 2017; Du et al., 2017; Chen et al., 2012; Ladell et al., 2013; Brumme et al., 2009; John et al., 2010).

Despite robust associations, translation into routine clinical decision-making is limited in the era of universal antiretroviral therapy (Ekenberg et al., 2019). Natural-history endpoints are modified by treatment, and heterogeneity in endpoints, population structure, viral subtype, and treatment era complicates direct bedside application. Current clinical actionability remains limited: despite being the strongest and most mechanistically mature HLA–infection association, HIV-control phenotypes such as elite control, viral-load set point, and disease progression do not currently translate into a validated individual-level HLA testing pathway, and the main translational value remains in mechanistic stratification, immunogen design, and interpretation of host-response heterogeneity. Where translation holds the greatest promise is population-level: HLA-restricted immunodominant epitopes inform vaccine immunogen design and T-cell-based strategies (Limou and Zagury, 2013). Additional replicated HLA associations are summarized in Table 4.

**TABLE 4:** Selected replicated HLA and HLA-region associations with infection outcomes and vaccine immunogenicity.

Infection/Vaccine	Endpoint	HLA allele/variant	Quantitative anchor	Evidence & population	Key references
HIV-1 infection	Durable host control (controllers vs. progressors)	HLA-B*57:03 (class I)	OR 5.1; P = 3.4 × 10^−18^	Fine-mapping/classical HLA typing at 4-digit resolution in African American controllers and progressors (n = 1,107); population-structure adjusted	McLaren et al. (2012)
HIV-1 infection	Viral-load set point (asymptomatic period)	HLA-B*57:01 tag (HLA-B*5701) SNP within HCP5 (endogenous retroviral element)	Explains 9.6% of variation in set-point viral load	Whole-genome association study; signal mapped to MHC region and associated with HLA-B*5701	Fellay et al. (2007)
HCV infection	Spontaneous viral clearance (single-source anti-D outbreak)	HLA-DRB1*01 (class II)	OR 4.9; uncorrected p = 0.001; pc = 0.01 (27.4% vs. 7.1%)	Single-source Irish anti-D cohort (n = 157 women), genotype 1b exposure; minimizes exposure/viral diversity confounding	Barrett et al. (1999)
HCV infection	Spontaneous viral clearance (meta-analysis)	HLA-DQB1*03:01 (class II)	OR 2.36 (1.62–3.43); P < 0.00001	Meta-analysis of 11 studies of spontaneous HCV clearance	Hong et al. (2005)
HBV vaccine (recombinant HBsAg)	Non-response to booster HB vaccination	HLA-DRB1 region SNP rs477515 (∼12 kb upstream of HLA-DRB1)	P = 4.81 × 10^−8^ (GWAS); P_combined = 3.98 × 10^−13^–1.42 × 10^−8^ (replication SNPs)	Chinese Han GWAS: 108 high-responders vs. 77 booster non-responders; validated in 3 additional cohorts (1,336 high-responders; 420 non-responders)	Pan et al. (2014)
HBV vaccine (recombinant HBsAg)	Antibody response (meta-analysis in healthy people)	HLA-DRB1*07 (class II)	Pooled OR 0.24 (reduced antibody response)	Meta-analysis of 15 studies (total n = 2,308) assessing DRB1/DQB1 alleles and vaccine antibody response	Li et al. (2013)
HBV vaccine (recombinant HBsAg)	Antibody response (meta-analysis in healthy people)	HLA-DRB1*13:01 (DRB1*1301) (class II)	Pooled OR 5.94 (increased antibody response)	Same meta-analysis (15 studies; n = 2,308)	Li et al. (2013)
HBV vaccine (recombinant HBsAg)	Antibody response (meta-analysis in healthy people)	HLA-DQB1*06:02 (class II)	Pooled OR 3.32 (increased antibody response)	Same meta-analysis (15 studies; n = 2,308)	Li et al. (2013)
HBV vaccine (recombinant HBsAg)	Antibody response (meta-analysis in healthy people)	HLA-DQB1*02 (class II)	Pooled OR 0.27 (reduced antibody response)	Same meta-analysis (15 studies; n = 2,308)	Li et al. (2013)
HBV vaccine (recombinant HBsAg)	Antibody response (meta-analysis of DPB1)	HLA-DPB1*02:02 (class II)	Pooled OR 4.53 (increased antibody response)	Systematic review/meta-analysis (studies through 1 June 2020)	Ou et al. (2021)
HBV vaccine (recombinant HBsAg)	Antibody response (meta-analysis of DPB1)	HLA-DPB1*04:01 (class II)	Pooled OR 3.33 (increased antibody response)	Same DPB1 meta-analysis	Ou et al. (2021)
HBV vaccine (recombinant HBsAg)	Antibody response (meta-analysis of DPB1)	HLA-DPB1*04:02 (class II)	Pooled OR 4.20 (increased antibody response)	Same DPB1 meta-analysis	Ou et al. (2021)
Conditionally informative: SARS-CoV-2 vaccination	Anti-RBD antibody levels + breakthrough infection risk	HLA-DQB1*06 (class II)	Anti-RBD: P = 3.2 × 10^−9^ (replicated); breakthrough: HR 0.63 (0.42–0.93), P = 0.02	Vaccine trial participants (n = 1,076) with replication in 1,677 vaccinees; ancestral/Alpha waves for breakthrough endpoint	Mentzer et al. (2022)
Conditionally informative: SARS-CoV-2 vaccination	IgG serostatus after first BNT162b2 dose	HLA-DRB1*13:02 (class II)	OR 0.75 (against IgG seronegativity); p = 2.34 × 10^−16^	United Kingdom Biobank GWAS (n = 54,066); structural/functional inference implicating DRβ1 position 71 in peptide-binding groove	Bian et al. (2024)

Legend. This author-defined table summarizes selected replicated HLA and HLA-region associations with infection outcomes and vaccine immunogenicity. Associations were author-selected if they met the following criteria: (i) effect size OR ≥ 2.0 or OR ≤ 0.5, or genome-wide significance in GWAS (P < 5 × 10^−8^) with independent replication; and (ii) at least partial mechanistic plausibility (e.g., peptide presentation/peptide-binding groove effects). Quantitative anchors are reported as stated in the cited sources (no back-calculation). For vaccine rows, odds-ratio direction follows the original source definition (e.g., antibody response vs. non-response) and is therefore not harmonized across studies. The table includes additional selected replicated associations beyond those highlighted in the main text. Rows are presented to distinguish higher-confidence replicated associations from conditionally informative exemplars. SARS-CoV-2 vaccine response is included in the latter category rather than as a routine-clinical HLA association. Most associations in this table should be interpreted as biologically informative or conditionally informative rather than as routine-testing candidates, unless explicitly stated otherwise in the main text. Abbreviations: OR, odds ratio; HR, hazard ratio; GWAS, genome-wide association study; MHC, major histocompatibility complex; SNP, single-nucleotide polymorphism; RBD, receptor-binding domain; HBsAg, hepatitis B surface antigen; pc, corrected P value.

#### 3.2.2 HCV

The strongest and most replicated HLA associations in hepatitis C virus (HCV) infection involve class II alleles and spontaneous viral clearance—the elimination of HCV RNA without antiviral treatment—which is generally estimated to occur in a substantial minority of acutely infected individuals, with reported rates varying by cohort and ascertainment (Thomas et al., 2009; Duggal et al., 2013; Hong et al., 2005; Gauthiez et al., 2017). A meta-analysis of 11 studies found that carriage of HLA-DQB1*03:01 and HLA-DRB1*11:01 approximately doubled the odds of spontaneous clearance (OR = 2.36, 95% CI 1.62–3.43, P < 0.00001; and OR = 2.02, 95% CI 1.56–2.62, P < 0.00001, respectively) (Hong et al., 2005). A larger systematic review encompassing 27 HLA studies confirmed HLA-DQB1*03, HLA-DRB1*04, and HLA-DRB1*11 among the most consistently associated class II signals, while also highlighting heterogeneity in design and endpoint definitions across the candidate-gene era (Gauthiez et al., 2017). These associations are biologically coherent for pharmacologists: HLA class II molecules shape the breadth and durability of CD4^+^ T helper responses during acute infection, and sustained, polyfunctional helper immunity is a recurring feature of individuals who clear HCV.

The Irish anti-D cohort provides particularly clean evidence. In this single-source outbreak, women were infected with a single HCV genotype 1b strain via contaminated anti-D immunoglobulin, minimizing viral diversity and route-of-exposure confounding that complicate most host genetic studies. Barrett et al. (1999) found that HLA-DRB1*01 was fourfold more frequent among the 73 spontaneous clearers than the 84 chronically infected women (27.4% vs. 7.1%, OR = 4.9, pc = 0.01). A second analysis of the same outbreak noted that the HLA-DRB1*01 association did not survive Bonferroni correction for multiple comparisons (Fanning et al., 2000). However, a subsequent multivariable analysis extending the Irish cohort to 319 women and incorporating an independent Swiss cohort (n = 461) confirmed HLA-DRB1*01:01 as independently protective (OR = 0.2, 95% CI 0.07–0.61, P = 0.005), with additive contributions from HLA-B*27 and the IFNL3 CC genotype (Fitzmaurice et al., 2015). Cross-ancestry replication further supports class II involvement: HLA-DRB1*11:01 and HLA-DQB1*03:01 were independently associated with clearance in Chinese Han and Li ethnic populations after adjustment for IFNL3 genotype (Huang et al., 2016; Huang et al., 2019). Among class I signals, HLA-B*57 has been associated with higher clearance rates (RR = 2.0, 95% CI 1.2–3.4), with evidence of viral immune escape within B57-restricted epitopes supporting a CD8^+^ mechanism (Kim et al., 2011). DQB1*05:01 has been reported within haplotype analyses of the anti-D cohort (Fanning et al., 2000) but has not been consistently replicated as an independent signal.

Host genetics in HCV reflects a hierarchy of effects: non-HLA IFNL3/IL28B-region variation is a dominant determinant of spontaneous clearance and helps explain cross-cohort heterogeneity in apparent HLA effects (Thomas et al., 2009; Heim et al., 2016). With direct-acting antivirals achieving high cure rates, routine HLA-based individual risk stratification is not established; the most realistic translational role for HLA remains mechanistic and population-level, including epitope selection and vaccine immunogen design (McConnell and Lim, 2018).

Accordingly, current clinical actionability remains limited because translation is constrained by retrospective clearance endpoints, cohort heterogeneity, ancestry-dependent effects, and the absence of an established genotype-guided intervention pathway in contemporary clinical practice.

#### 3.2.3 Dengue

Dengue virus infection spans a spectrum from self-limited disease to severe dengue, with risk strongly shaped by immune history (including secondary infection), infecting serotype, and host factors. Across studies, recurrent signals have been reported for HLA-A*24 and HLA-DRB1*11 as risk alleles and HLA-A*33 as protective in some Asian and Southeast Asian datasets, with additional protective signals suggested for HLA-B*44 (Chen et al., 2019; Ghosh et al., 2025). However, findings are inconsistent and often population specific, plausibly reflecting differences in ancestry structure, secondary infection status, serotype distribution, phenotype definition, typing resolution (Stephens et al., 2002; Alshammari et al., 2024). In dengue, HLA class I expression may also restrain innate effector activity: blocking HLA class I on DENV-infected monocytes nearly doubled the frequency of CD107a+ NK cells in one study, supporting an HLA–KIR contribution to early antiviral control and immunopathology (McKechnie et al., 2019). Overall, HLA associations in dengue remain biologically interesting but are not clinically actionable for individual risk stratification at present.

#### 3.2.4 SARS-CoV-2/COVID-19

A large number of studies have examined HLA variation in SARS-CoV-2 susceptibility, COVID-19 severity, and immune response, but the literature has not converged on robust, transportable signals. Across cohorts, many nominal associations have been reported, yet direction and magnitude often vary across populations and endpoints, with limited independent replication (Deb et al., 2022; Dobrijević et al., 2022). Inconsistency is driven by heterogeneous endpoint definitions, variable typing and imputation resolution, population stratification, evolving variants, and vaccination- and treatment-era confounding (Castro-Santos et al., 2023). Accordingly, HLA does not currently support clinically useful COVID-19 risk stratification for routine practice.

Together with dengue, COVID-19 illustrates how biologically plausible HLA associations can accumulate without becoming transportable or decision-grade when endpoints and populations are heterogeneous (Deb et al., 2022; Dobrijević et al., 2022; Castro-Santos et al., 2023).

#### 3.2.5 Vaccine responses: HLA associations with immunogenicity and non-response

The hepatitis B vaccine provides the strongest and most extensively replicated evidence for HLA-restricted variation in vaccine immunogenicity. Approximately 5%–10% of healthy adults fail to produce protective levels of antibody following a standard vaccination course (Pan et al., 2014). A meta-analysis of 15 studies encompassing 2,308 subjects found that carriage of DRB1*07 was associated with a markedly reduced likelihood of seroconversion (pooled OR 0.24 for antibody response), while DRB1*01 (OR 2.73), DRB1*13:01 (OR 5.94), and DRB1*15 (OR 2.29) were each associated with significantly increased antibody response (Li et al., 2013). A cross-vaccine meta-analysis of 11,686 subjects confirmed these findings and additionally identified DQA1*02:01 (OR = 2.21) and DQB1*02:01 (OR = 2.03) as risk alleles for non-response to hepatitis B vaccination (Posteraro et al., 2014). A separate meta-analysis of the DPB1 locus reported even larger effect sizes: DPB1*02:02 (OR = 4.53) and DPB1*04:02 (OR = 4.20) were associated with enhanced response (Ou et al., 2021). A genome-wide association study in Chinese Han populations localized the genetic signal to SNPs upstream of DRB1 at genome-wide significance (P = 4.81 × 10^-8), validated across three independent replication cohorts totaling 1,756 subjects (Pan et al., 2014). These statistical associations have mechanistic support: peptide-binding studies demonstrated that the HLA-DR7 molecule encoded by DRB1*07:01 binds fewer high-affinity peptides from the hepatitis B surface antigen than DR1 or DR13, directly impairing CD4^+^ T helper cell activation required for antibody production (Godkin et al., 2005). The DRB1*07–DQB1*02:01 haplotype thus represents arguably the best-characterized example of an HLA-restricted vaccine failure in clinical medicine. Cohort evidence also supports independent HLA contributions to hepatitis B vaccine response alongside cytokine variation (Wang et al., 2004).

Influenza vaccine responses also show HLA class II modulation, although the evidence base is smaller and less consistently replicated. In a cohort of 73 at-risk adults, DRB1*07-positive individuals were significantly overrepresented among non-responders to trivalent subunit influenza vaccine as measured by hemagglutination inhibition titers at 28 days (13/32 non-responders vs. 6/41 responders; P = 0.016), while DQB1*06:03-9/14 carriage was associated with response (P = 0.0045) (Gelder et al., 2002). Broader immunogenetic analyses have confirmed that HLA class II and cytokine gene polymorphisms contribute to the inter-individual variability in humoral responses to inactivated influenza vaccines, but no single allele has been replicated with the consistency seen for hepatitis B (Poland et al., 2008).

HLA associations have also been reported for live attenuated childhood vaccines, with replication across measles vaccine cohorts (Ovsyannikova et al., 2012; Haslund et al., 2023).

For SARS-CoV-2 vaccines, emerging GWAS data have identified reproducible HLA class II signals. In ChAdOx1 nCoV-19 trial participants (n = 1,076, replicated in 1,677), HLA-DQB1*06 reached genome-wide significance for higher anti-receptor-binding-domain antibody at 28 days post-first dose (P = 3.2 × 10^-9), and carriers had a lower risk of PCR-confirmed breakthrough infection (HR = 0.63, 95% CI 0.42–0.93) (Mentzer et al., 2022). A United Kingdom Biobank GWAS (n = 54,066) found HLA-DRB1*13:02 most significantly associated with IgG seropositivity after the first BNT162b2 dose (OR = 0.75 against seronegativity, P = 2.34 × 10^-16), with a structural mechanism involving amino acid position 71 in the peptide-binding groove (Bian et al., 2024). An independent population-based study of 357,806 vaccinated individuals confirmed that DQB1*06 alleles were linked to improved antibody responses, though the combined effect of HLA alleles on breakthrough infection risk was characterized as modest (Xie et al., 2024).

From a clinical-actionability standpoint, hepatitis B vaccine response remains the strongest and most clearly replicated HLA–vaccine association identified to date. The DRB1*07–DQB1*02:01 non-response haplotype and response-associated class II signals have sufficient effect size, replication, and mechanistic plausibility to justify inclusion among robust pharmacogenomic findings. By contrast, the emerging SARS-CoV-2 DQB1*06 and DRB1*13:02 signals are best regarded as conditionally informative: they are scientifically interesting and replicated in some settings, but still require further characterization of clinical impact across diverse populations, time-from-vaccination windows, and evolving vaccine platforms. Outside hepatitis B, routine HLA-guided vaccination or booster strategies are not established in clinical practice, and most HLA–vaccine findings remain insufficient for individual-level risk stratification. Additional selected HLA–vaccine associations are summarized in Table 4 (Li et al., 2013; Pan et al., 2014; Mentzer et al., 2022; Bian et al., 2024).

### 3.3 Implementation and limitations

The preceding sections illustrate a striking asymmetry: while HLA pharmacogenomics has been successfully implemented in clinical practice for drug hypersensitivity prevention, none of the infection-outcome associations reviewed here currently supports routine individual-level HLA testing in clinical practice, and hepatitis B vaccine non-response remains the closest vaccine-related example to practical translational relevance. Understanding why clarifies both current limitations and realistic prospects for this field (Manson et al., 2020; Negrini and Becquemont, 2017b).

Several biological and methodological features of infectious diseases dilute and confound HLA signals. Infection outcomes are shaped by pathogen diversity (serotypes, genotypes, and immune escape variants), co-infections, prior immunity, treatment history, and host factors such as immune status and comorbidities; social and socioeconomic determinants influence exposure risk and care pathways. Endpoints are also seldom binary and are frequently heterogeneous across cohorts: “COVID-19 severity,” “HIV progression,” and “severe dengue” may be defined using different clinical thresholds, time windows, and evolving classification systems (as discussed in Sections 3.2.3–3.2.4). Exposure misclassification and treatment-era confounding (vaccination, evolving therapeutics, and changing standards of care) further undermine replicability across studies. Interpretation of HLA associations also depends on typing resolution and on additional modifiers beyond HLA itself, including T-cell receptor repertoire, immune status, concomitant infections, and factors influencing drug exposure (Negrini and Becquemont, 2017b; Castro-Santos et al., 2023; Heim et al., 2016).

Despite these barriers, HLA–infection research retains practical value in two domains. The first is vaccine immunogen design: identifying HLA-restricted epitopes and optimizing HLA supertype coverage can maximize population-level T-cell immunity for targets such as HIV and HCV, without requiring individual-level risk prediction (Parvizpour et al., 2020; MacDonald et al., 2001). The second, more limited niche is risk stratification in selected high-stakes vaccine outcomes where effects are strong, replicated, and mechanistically supported—most notably hepatitis B vaccine non-response linked to DRB1*07–DQB1*02:01 (Section 3.2.5). Even here, translation into routine practice would require evidence that genotype-guided strategies improve net outcomes and are operationally and economically justified, given the typically modest positive predictive value of single alleles.

Taken together, these findings indicate that HLA associations in infection and vaccine outcomes are currently most useful for biological stratification, epitope selection, and vaccine design rather than routine bedside genotyping; the main barriers to individual-level translation are endpoint heterogeneity, ancestry dependence, confounding, modest predictive performance, and the lack of validated genotype-guided intervention pathways (Pan et al., 2014; Parvizpour et al., 2020).

## 4 Discussion

The evidence synthesized in this review indicates that HLA polymorphisms are not merely markers of immune diversity, but clinically meaningful modifiers of therapeutic variability in infectious diseases. Across severe anti-infective drug toxicity, infection outcomes, and vaccine responsiveness, the same biological axis repeatedly emerges: variation in antigen presentation helps explain why apparently similar exposures can result in markedly different clinical trajectories. These two domains represent distinct clinical expressions of a shared host immunopharmacological architecture (Pirmohamed et al., 2015).

In clinical practice, the most established application is still the prevention of severe HLA-linked adverse drug reactions. Abacavir provides the clearest proof of principle, not simply because the association with HLA-B*57:01 is strong, but because genotype, phenotype, and therapeutic choice align unusually well: the reaction is clinically recognizable, the risk is avoidable before exposure, and effective alternatives are available (Mallal et al., 2008; Martin et al., 2014). Dapsone illustrates the same logic in a different anti-infective setting and shows that implementation may still be justified even when predictive performance is incomplete, provided the prevented event is serious and therapeutic substitution is feasible (Zhang et al., 2013; Liu et al., 2019). Flucloxacillin, by contrast, makes the opposite point: even a striking HLA signal does not automatically justify screening when the underlying phenotype is too rare for routine testing to yield meaningful preventive value (Daly et al., 2009; Nicoletti et al., 2019). Clinical utility emerges when genetic signal, phenotype precision, and therapeutic consequences converge.

A key lesson here is the difference between finding an association and showing that it can actually guide decisions. Relative effect size alone is never sufficient. Across anti-infective HLA associations, directly comparable absolute-risk estimates are not uniformly available across drugs and phenotypes; for many drug-allele pairs, the most transportable measures remain effect estimates and test characteristics rather than cross-condition prevalence comparisons (Manson et al., 2020; Negrini and Becquemont, 2017b). What ultimately matters is whether a genotype changes what can reasonably be done—whether it supports avoidance, substitution, closer monitoring, or a more informed estimate of benefit–risk. In that sense, HLA-associated anti-infective toxicity offers more than successful examples in pharmacogenetics; it provides a model for how host genomics becomes clinically relevant only when biological signal, phenotype clarity, and therapeutic choice intersect. It also explains why some biologically persuasive associations remain translationally immature: screening readiness depends not only on the reality of the signal, but on timing, absolute risk, local allele frequency, and the practical consequences of excluding carriers from first-line therapy (Pirmohamed et al., 2015; Manson et al., 2020).

The infection and vaccine pillar shows a different, and in some respects more nuanced, form of importance. Here, HLA often provides substantial biological insight, but less immediate therapeutic leverage. HIV remains the clearest example that HLA class I variation can shape clinically meaningful control of infection (Fellay et al., 2007; McLaren et al., 2015), while the hepatitis B vaccine literature provides one of the strongest demonstrations that HLA class II variation contributes to reproducible differences in immunogenicity (Li et al., 2013; Pan et al., 2014). Yet even in these best-supported settings, the path to implementation is less direct than in drug hypersensitivity. Infection and vaccine outcomes are shaped by a more layered interaction among pathogen diversity, immune history, treatment context, comorbidities, ancestry, and environmental exposure. As a result, HLA may be highly informative without being immediately deployable as an individual-level clinical test. Its value often lies less in bedside prediction than in clarifying host-response heterogeneity, refining mechanistic interpretation, and informing trial design, immunogen selection, or vaccine development.

The practical question now is which HLA signals are strong and consistent enough to influence treatment decisions, rather than whether HLA matters at all. In the adverse drug reaction space, that means prospective validation, real-world estimates of predictive performance, and clearer definitions of net clinical benefit once alternatives, turnaround time, and health-system constraints are taken into account. In infection and vaccine research, it means cleaner endpoints, broader ancestral representation, and more integrated models in which HLA is interpreted alongside non-HLA host factors rather than in isolation. This also has implications for pre-emptive pharmacogenomic implementation: where broader pharmacogenetic panels are being introduced, HLA markers are likely to be most useful when linked to severe, preventable phenotypes and embedded within decision-oriented workflows rather than treated as stand-alone signals (Peruzzi et al., 2023; Tamraz et al., 2025). The more important question is when that influence becomes sufficiently stable, interpretable, and decision-relevant to improve practice.

In both areas, careful interpretation mainly comes down to how solid the phenotype definition is and which populations are being studied. Phenotype definition is a major determinant of apparent effect size, and HLA allele frequencies and linkage patterns vary across ancestries with direct implications for transferability and predictive performance (Luczak et al., 2021; Luo et al., 2021). Mechanistic support is also uneven: some associations are underpinned by coherent structural and functional data, whereas others remain statistically persuasive but biologically less resolved.
